# Rheological, mechanical, and cytotoxic properties of sustainable NR/glass fiber composites: prospects for advanced applications

**DOI:** 10.1186/s13065-025-01676-y

**Published:** 2025-11-24

**Authors:** Doaa S. Mahmoud, Abdelmohsen M. Soliman, Fahima M. Helaly, Salwa H. El-Sabbagh

**Affiliations:** 1 Polymers and Pigments Department, National Research Centre, Giza, Egypt; 2 Therapeutic Chemistry Department, National Research Centre, Giza, Egypt

**Keywords:** Glass fiber (GF), Mechanical properties, Natural rubber (NR), Rheological characteristic, Swelling resistance

## Abstract

Glass fiber is an inorganic reinforcement material widely used in rubber-based technological products. This study investigates the properties of natural rubber/glass fiber composites, focusing on their potential to enhance mechanical performance while reducing costs. A series of tests were conducted to evaluate the rheological, mechanical, swelling, and morphological characteristics of the investigated natural rubber composites. Quantitative analysis showed that the composition containing 16 phr glass fiber resulted in a 59.5% increase in tensile strength compared to composites filled with traditional fillers such as sodium bentonite, calcium carbonate, silica, and calcined kaolin. The composite also showed a significant boost in mechanical properties, with notable increases in modulus at 100% and 300% strain, strain energy, and a stronger Payne effect, which indicates strong interfacial adhesion, as confirmed by morphological analysis. Morphological analysis of fractured surfaces confirmed excellent adhesion between glass fiber and natural rubber. Notably, the natural rubber/16 phr glass fiber composite exhibited exceptional swelling resistance in toluene, with a quantifiable reduction in swelling compared to other composites. This makes it suitable for automotive interiors. Cytotoxicity tests verified that the material is non-toxic, supporting its use in human-related applications. These results suggest that glass fiber is a better and more sustainable reinforcing filler for enhancing the performance of rubber products in the automotive industry.

**Graphical Abstract:**

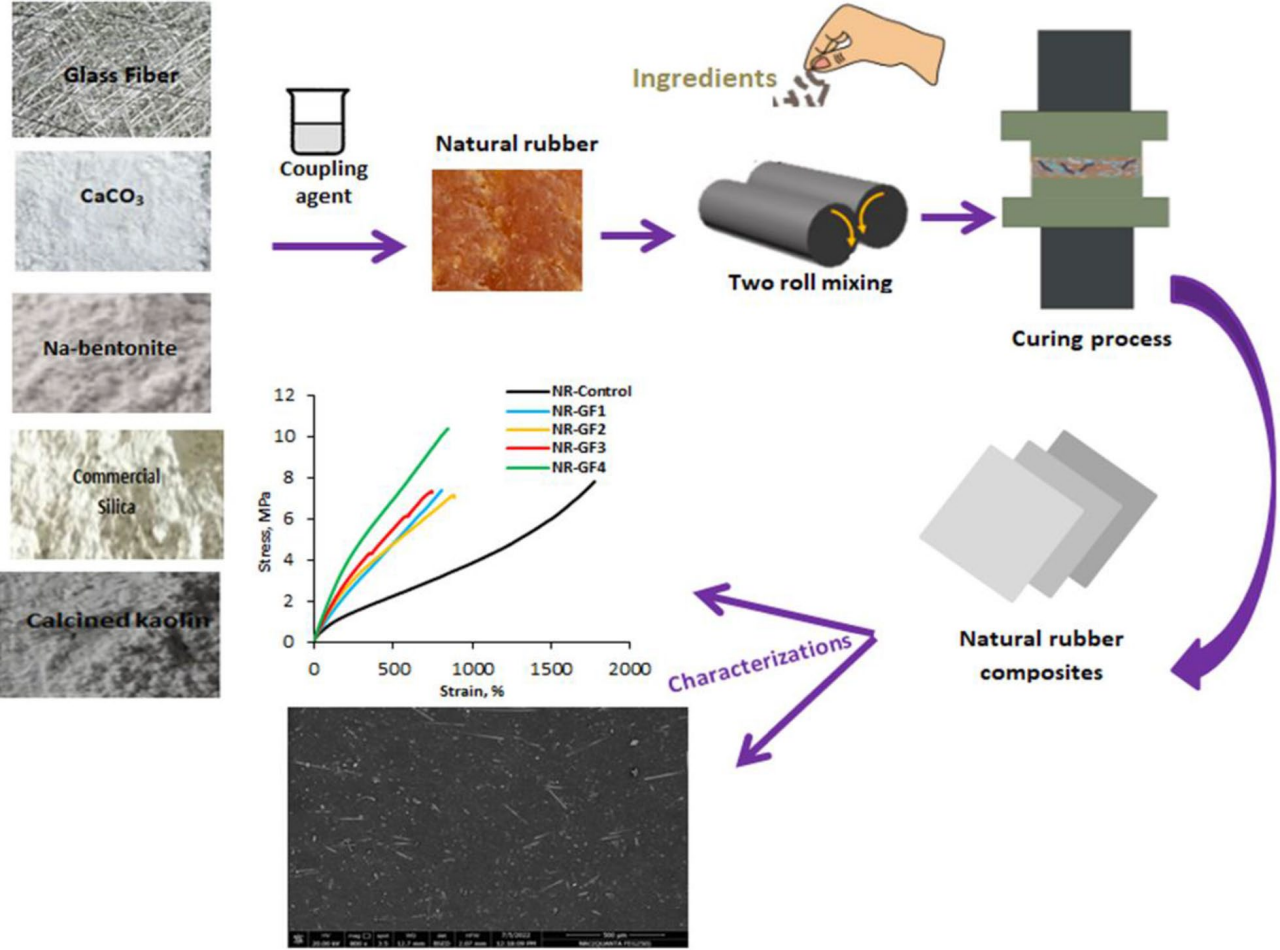

## Introduction

Advanced composite materials are at the forefront of technological innovation, offering enhanced performance for use in aerospace, automotive, wastewater treatment, and high-end industrial applications [1]. In recent years, rubber composites have gained significant global research attention and development due to their favorable properties, including low corrosion, cost efficiency, ease of maintenance, and design variability. Rubber materials are distinguished by their optimal combination of mechanical robustness, structural stiffness, and light weight.

These engineered rubber materials, which combine rubber with reinforcing agents such as fabric or metal, exhibit enhanced mechanical strength and performance across a wide range of industrial applications. The automotive industry extensively employs rubber in the fabrication of components like tires, sealing elements, flexible hoses, transmission belts, and suspension parts [2]. Natural rubber (NR) is one of the most commonly used rubber materials, known for its unique set of properties and benefits. It exhibits high tensile strength, superior airtightness, excellent crack resistance, and good elasticity. However, its aging resistance is relatively poor, primarily due to the high content of double bonds in its molecular structure [3, 4]. The performance of natural rubber-based composites is largely determined by reinforcement scale (micro/nano), processing parameters, filler dispersion, interfacial interactions, and the resulting morphology.

In order to achieve improved mechanical properties at a reasonable cost, NR composites utilize reinforcing fillers. Fillers significantly influence the viscoelastic properties of rubber composites by enhancing strain-induced crystallization and damping behavior. They also extend the linear viscoelastic range and are essential for tuning elastomer performance. Key filler characteristics such as particle size, structure, and surface chemistry affect durability and mechanical properties. Carbon black remains the standard filler in automotive applications due to its low cost and effectiveness; rising demand and limited supply present future availability concerns [5]. Szadkowski et al. [6] found that phytic acid’s phosphorus structure reduces flammability in NR composites, enhancing fire safety. Its combination with silanized mineral fillers improves fire resistance without compromising mechanical or rheological properties, offering a sustainable solution for various uses. Manaila et al. [7] developed an innovative elastomeric material based on natural rubber (NR) by replacing conventional fillers such as carbon black and silica with natural hemp fibers. They observed that increasing the fiber content led to higher hardness, tensile strength, and tear strength, suggesting that the strong interaction between the hemp fibers and NR provides a reinforcing effect. Suppanucroa et al. [8] discovered that NR sponges reinforced with 45 phr cellulose fibers and 1 phr sodium alginate (SA) demonstrated compressive stress and elasticity modulus values approximately 5.0–6.8 times greater than those of the unmodified NR sponge. NR-cellulose fiber sponges exhibited favorable physical properties and demonstrated enhanced biodegradability under natural soil conditions. Fibrous reinforcements are highly favored due to their superior strength and stiffness. They effectively strengthen the matrix, thereby enhancing its properties as required [9, 10]. Synthetic fibers are generally more uniform, cost-effective, and predictable in performance. While glass fiber is often preferred in engineering applications, synthetic fibers can provide improved stiffness and toughness when combined with ductile matrices like metals or polymers [11, 12]. Glass fibers offer strong insulation, high strength, chemical and moisture resistance, and fire retardance, maintaining durability even in humid conditions [13]. Deshang et al. [14] investigated the behavior of glass fiber (GF)/rubber composites developed in terms of wear and friction during mixing processes. The data showed that the addition of 3 phr glass fiber (GF) was most effective in minimizing both abrasive and total wear; however, this amount of GF also resulted in increased corrosive wear due to larger silanization reactions. Larger amounts of GF (5–7 phr) led to significantly higher levels of all wear types and surface roughness, indicating a damaging effect on both the material and equipment. Wang et al. [15] produced NBR/GF composites using mechanical blending. They observed that increasing the amount of GF led to a progressive reduction of the vulcanization rate and that enhancement of the tensile strength and stress at 100% elongation was observed initially with increasing GF content, but both properties began to decrease beyond a certain threshold.

The advancement of NR/glass fiber composites has served as a key inspiration for researchers and has created valuable opportunities to enhance the quality of life worldwide [16]. Research on rubber composites for automotive applications offers novel perspectives and methodologies for creating composite materials with exceptional mechanical strength, low weight, and swelling resistance. Fiber-reinforced elastomer composites have received significant attention due to their potential to combine flexible mechanical reinforcement with flexural compliance. However, achieving high performance in these composites is challenging due to fiber-rubber interfacial adhesion, filler incorporation, and the impacts of the fibers on the network structure during curing. Despite the considerable amount of research published on NR/fiber composites, limited studies have examined the use of glass fibers as fillers and their effects on cure kinetics, network structure, and durability. This study aims to prepare and assess natural rubber (NR) composites reinforced with glass fiber, with a focus on analyzing their rheological, mechanical, and morphological properties. Additionally, the study will investigate the cytotoxicity effects on normal human cell lines. The composites will be formulated with varying concentrations of GF, calcium carbonate (CaCO_**3**_), sodium bentonite (Nb), silica (Si), and calcined kaolin (CK) to evaluate their performance and biocompatibility.

## Materials and methods

### Materials

Glass fibers (GF) are silica-based, with diameters approximately 10–12 μm and composed of about 50–60% SiO₂ and various other oxides, including calcium, boron, sodium, aluminum, and iron. These fibers were obtained from the Jushi Group located in Suez, Egypt. Type SMR-20 natural rubber (NR), which contains 95–99% cis-1,4-polyisoprene with a density of 0.931, Mooney viscosity ML (1 + 4) = 60–90 at 100 °C, and glass transition temperature Tg =  − 75 °C was graciously provided by Transport and Engineering Company (TRENCO), Alexandria. The components needed to vulcanize rubber, such as Zinc Oxide (ZnO) (purity: 99.9%), Stearic Acid (SA) (purity: 98.5%), aromatic oil (naphthenic processing oil), and Sulfur (S), were all obtained from Aldrich Company, Germany. N-Cyclohexyl-2-benzothiazole sulphenamide (CBS) (purity: 98%) was obtained from Rheinehemie, Germany. Polymerized 2, 2, 4-trimethyl-1, 2-dihydroquinoline (TMQ) is a commercial-grade product. Bentonite, Sodium (Nb) (purity: 99%) (Alfa Aesar and Co. (Kandel, Germany)), Calcium carbonate (CaCO₃) from the Egyptian Jordan Company for Carbonate, hydrated silica (Hi-Si) from PPG Industries Inc. (Delfzijl, Netherlands), and kaolin processed from Egypt. All materials are commercial grade products. The purity of (3-Aminopropyl) trimethoxysilane from Alfa Aesar (Kandel, Germany) is usually 97% when applied as a coupling agent.

### Methods

#### Thermal treatment of kaolin

To obtain metakaolinite, Egyptian kaolin was calcined in a muffle furnace for 120 min at 600 °C. Equation (1) illustrates how the amorphous phase known as metakaolinite is produced when kaolinite undergoes dehydroxylation during calcination [17, 18]. 200 g of metakaolinite was treated with an NaOH solution and then allowed to react for 120 min at 600° C in a muffle furnace. After 24 h of ageing, the liquid and solid phases were separated. The solid phase was washed twice with water, centrifuged, then dried for 8 h at 100–110 °C [19].

1 $${Al}_{2}{O}_{3}.2{SiO}_{2}.2{H}_{2}O \left(kaolinite\right)\stackrel{\Delta \left(600^\circ C\right)}{\to }{Al}_{2}{O}_{3}.2{SiO}_{2}\left(metakaolinite\right)+2{H}_{2}O$$

#### Preparation of composites

**Fig. 1:**
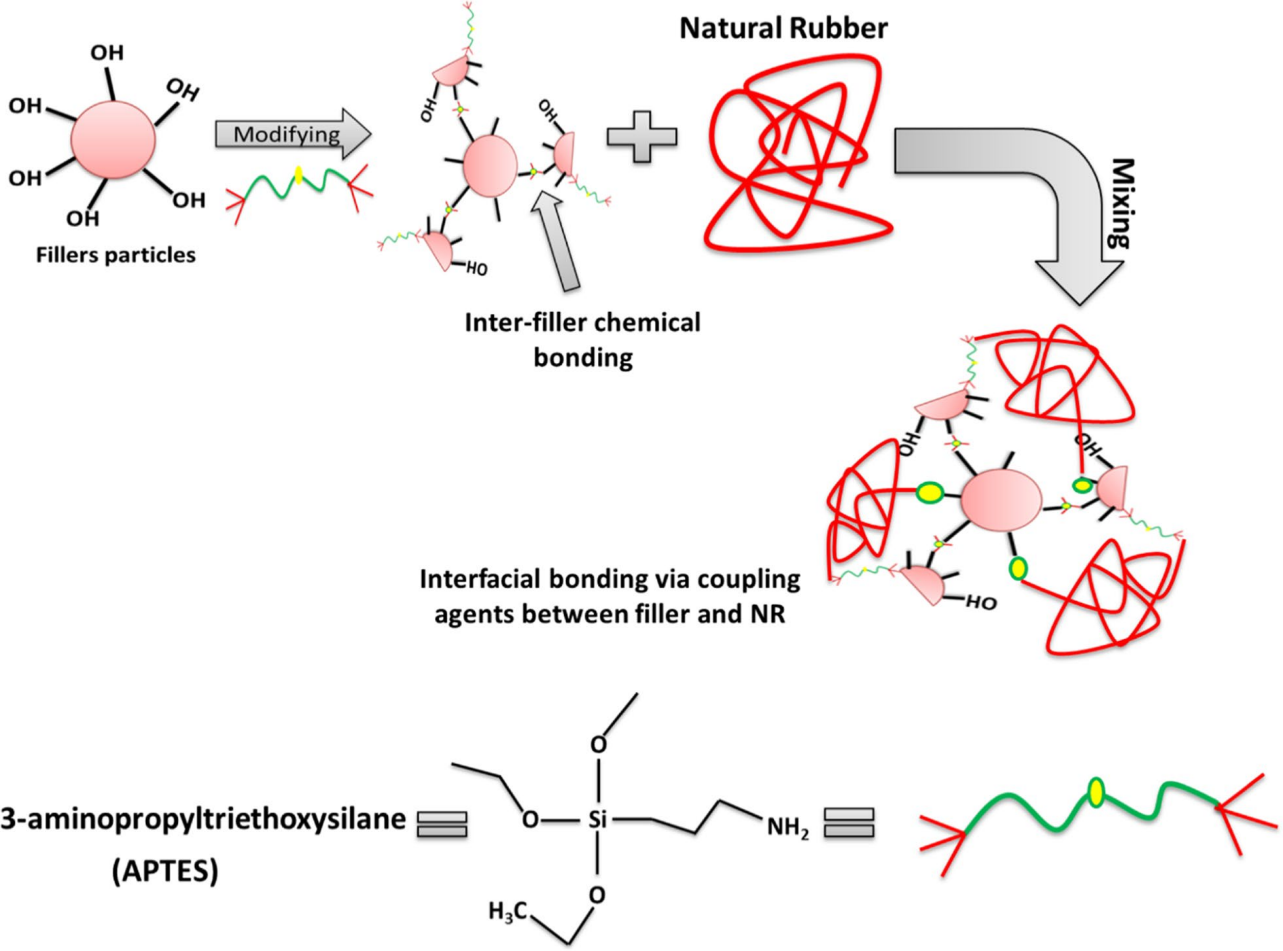
Schematic illustration of the mechanism for the formation of the proposed NR composites

At room temperature (28 ºC), rubber compounds were manufactured in an open two-roll mill. Natural rubber was first introduced into the two-roll mill, where it was compressed and molded into a thin, smooth rubber sheet around one of the rolls while it cycled at 24 rpm for 4–5 min. Ingredients were incorporated in the order in which they are present in Table 1. During compounding, varying amounts of GF, Nb, CaCO_**3**_, Si, and CK were added at two-minute intervals in the presence of a coupling agent (3-aminopropyl) trimethoxysilane (as shown in Fig. 1). The proposed mechanism for surface modification of filler particles with silane coupling agents is provided schematically in Fig. 1. Finally, for producing the mixed rubber, sulfur was added and mixed for two minutes. The sheets were heated electrically and vulcanized at 142 °C under pressure of 40 kg/cm^**2**^ for their respective cure times. Table 1 summarizes the rubber compound recipes.

**Table 1:** NR composites expressed in parts per hundred parts of rubber (phr)

Name of sample	Ingredients in Phr					
	Coupling agent (APTES)	Glass fiber	CaCO _**3**_	Na-bentonite	Silica	Calcined Kaolin
NR-control	–	–	–	–	–	–
NR-GF_1_	2	2	–	–	–	–
NR-GF_2_	2	4	–	–	–	–
NR-GF_3_	2	8	–	–	–	–
NR-GF_4_	2	16	–	–	–	–
NR-Ca_1_	2	–	2	–	–	–
NR-Ca_2_	2	–	4	–	–	–
NR-Ca_3_	2	–	8	–	–	–
NR-Ca_4_	2	–	16	–	–	–
NR-Nb_1_	2	–	–	2	–	–
NR-Nb_2_	2	–	–	4	–	–
NR-Nb_3_	2	–	–	8	–	–
NR-Nb_4_	2	–	–	16	–	–
NR-S_1_	2	–	–	–	2	–
NR-S_2_	2	–	–	–	4	–
NR-S_3_	2	–	–	–	8	–
NR-S_4_	2	–	–	–	16	–
NR-CK_1_	2	–	–	–	–	2
NR-CK_2_	2	–	–	–	–	4
NR-CK_3_	2	–	–	–	–	8
NR-CK_4_	2	–	–	–	–	16

Composition of typical elastomer mixture: NR rubber (100 phr); zinc oxide (5 phr); stearic acid (2.5 phr); fillers (2–20 phr); oil (2 phr); CBS (0.8 phr); TMQ (1 phr) and sulphur (2.5 phr)

### Measurements and analysis

#### Infrared spectroscopy

Fourier-transform infrared (FTIR) spectra of glass fiber were obtained after drying with a Joint Assault Signal Company FTIR-6100E, Japan.

#### SEM and EDS analysis

The surface morphology of GF along with the dispersion of GF and the different fillers in the NR matrix was examined with a Quanta FEG 250 scanning electron microscopy (SEM) coupled with energy-dispersive X-ray spectroscopy (EDS).

#### BET surface area and porosity analysis

The Brunauer–Emmett–Teller method was used to determine the specific surface area of solids and powders. NR specimens (1 × 1 × 1.5 mm) were dried under inert gas or vacuum to remove contaminants to obtain a pure sample before being cooled to cryogenic temperatures, allowing adsorption of a probe gas. The volume of the adsorbed gas corresponding to monolayer coverage was analyzed using BET theory, allowing calculation of surface area (m^2^/g). BET measurements were performed using a TriStar II 3020 Micromeritics analyzer, which provided the specific surface area, pore size distribution, and pore volume of the prepared sample.

#### Rheological characteristics

Moving Die Rheometer (Monsanto oscillating disc rheometer-100) was used to determine the curing parameters for processing of the composite samples as per ASTM D 2084–07, 2007a.

#### Mechanical properties

Physical characteristics were assessed using a tensile testing machine, Zwick Roell Z010 (Ulm, Germany), in compliance with ASTMD412-06a (2013).

#### Swelling resistance

The equilibrium swelling (EQs) tests were carried out in toluene as an organic solvent and water as stated by ASTM D3616-95 (2019). Square samples from the prepared investigated natural rubber composites containing various specific types of filler are weighed and soaked for one day in toluene and tap water at room temperature. The samples were weighed after soaking, and it is possible to compute the equilibrium swelling EQs% using the following equation [10]:

2 $$\text{EQs}= \frac{{\text{W}}_{\text{s}}-{\text{W}}_{\text{dr}}}{{\text{W}}_{\text{dr}}} \times 100$$

where W_**S**_ is the weight of the swelled specimen and W_**dr**_ is the weight of the dried specimen. The cross-link density (ν_**dens**_) is calculated using the Flory-Rehner equation [20, 21]:

3 $${\upnu }_{\text{dens}}=\frac{1}{{2\text{M}}_{\text{WC}}}$$

where M_**wc**_ is the molecular weight between two cross-links (g/mol)

4 $$ {\text{Mw}}_{{\text{c}}} = \frac{{ - \rho_{{\text{E}}} {\text{V}}_{{\text{t}}} ({\text{V}}_{{\text{r}}}^{1/3} )}}{{\ln (1 - {\text{V}}_{{\text{r}}} ) + {\text{V}}_{{\text{r}}} + {}_{\chi }{\text{V}}_{{\text{r}}}^{2} }} $$

where ρ_**E**_ is the density of rubber (NR is 0.931 g/cm^3^), toluene's molar volume (V_**t**_) is 106.35 cm^**3**^ mol^**−1**^, x is the rubber interaction factor (where the x of (NR) is 0.393), and V_**r**_ is the volume fraction of swelled rubber, which can be estimated from the mass and density of rubber samples and the solvent.

The Lorenz and Park equations [22] were utilized to study the rubber-filler interaction:

5 $$\frac{{\text{EQ}}_{\text{f}}}{{\text{EQ}}_{\text{NR}}}={\text{Ae}}^{-\text{Z}}+\text{B}$$

where EQ_**f**_ represents the filler's swelling value and EQ_**NR**_ represents the gum's swelling value. A and B are constants, while z is the filler weight ratio in the vulcanizate. The increased swelling ratio value ($\frac{{\text{EQ}}_{\text{f}}}{{\text{EQ}}_{\text{NR}}}$) suggests a less effective interaction between the filler under investigation and the NR rubber matrix. From the Flory–Huggins from, the change in elastic Gibbs free energy may be calculated [17]:

6 $$ \Delta {\text{G}} = {\text{RT}}[\ln (1 - {\text{V}}_{{\text{r}}} ) + {\text{V}}_{{\text{r}}} + {}^{\chi }{\text{V}}_{{\text{r}}}^{2} ] $$

where R is the general gas constant and T is the temperature in Kelvin (25 + 273); the amendment entropy ΔS can be computed using the statistical theory of rubber elasticity by [17]:

7 $$ \Delta {\text{S}} = - \frac{{\Delta {\text{G}}}}{{\text{T}}} $$

### Cytotoxic effect on human normal fibroblast cell line (BJ1):

The mitochondrial-dependent reduction of yellow MTT (3-(4,5-dimethylthiazol-2-yl)-2,5-diphenyl tetrazolium bromide) to purple formazan was used to assess cell viability [23]. Procedure: Using a Laminar flow cabinet biosafety class II (Baker, SG403INT, Sanford, ME, USA), all of the following operations were carried out in a sterile environment. The cells were cultured in DMEM-F12 media with 1% L-glutamine, 1% antibiotic–antimycotic mixture (10,000 µg/ml streptomycin sulfate, 10,000 U/ml potassium penicillin, and 25 µg/ml Amphotericin B) at 37ºC with 5% CO_**2**_. After batch culturing for ten days, the cells were seeded into 6-well microtiter plastic plates with fresh complete growth media at a concentration of 10 × 10^**6**^ cells/well. The plates were placed in a water-jacketed CO_**2**_ incubator (Sheldon, TC2323, Cornelius, OR, USA) and kept at 37⁰C for twenty-four hours under 5% CO_**2**_. After aspirating the media, fresh serum-free medium was added, and the cells were cultured with various concentrations of the test sample (10, 5, 2.5, and 1.25 mg/ml) or alone (negative control). Following 48 h of incubation, the medium and rubber were removed, and 600 ul of MTT solution (2.5 μg/ml) was added to each well. The wells were then incubated for an additional four hours at 37 °C with 5% CO_**2**_. Each well was filled with 3 mL of 10% sodium dodecyl sulphate (SDS) in deionized water, which was then incubated overnight at 37 °C to terminate the reaction and dissolve the crystals that had formed [24]. A microplate multi-well reader (Bio-Rad Laboratories Inc., model 3350, Hercules, California, USA) was then used to measure the absorbance at 595 nm with a reference wavelength of 620 nm [25, 26]. Statistical analysis was performed using the independent t-test in the SPSS 11 software, comparing the samples and the negative control (cells containing a vehicle). Plant extracts are dissolved in DMSO, and the final concentration of the extract on the cells was less than 0.2%. Using the following formula, the percentage change in viability was determined:((Reading of extract / Reading of negative control) − 1) × 100 The SPSS 11 program was used to do a probit analysis in order to determine the IC50 and IC90. This experiment was repeated three times.

Cell line: Human normal fibroblast cell line (BJ1) was obtained from American Type Culture Collection ATCC (Rockville, Maryland, USA). Cells were cultured in Eagle medium (IIET, Wroclaw, Poland) supplemented with 2 mM L-glutamine, 10% fetal bovine serum, 8 ug/mL of insulin and 1% mem non-essential amino acid solution 100x (all from Sigma–Aldrich Chemie GmbH, Steinheim, Germany). Culture media was also supplemented with antibiotics: 100 μg/ml streptomycin (Sigma–Aldrich Chemie GmbH, Steinheim, Germany) and 100 units/ml penicillin (PolfaTarchomin SA, Warsaw, Poland). All cell lines were grown at 37 °C with 5% CO2 humidified atmosphere.

## Results and discussion

### FTIR analysis

**Fig. 2:**
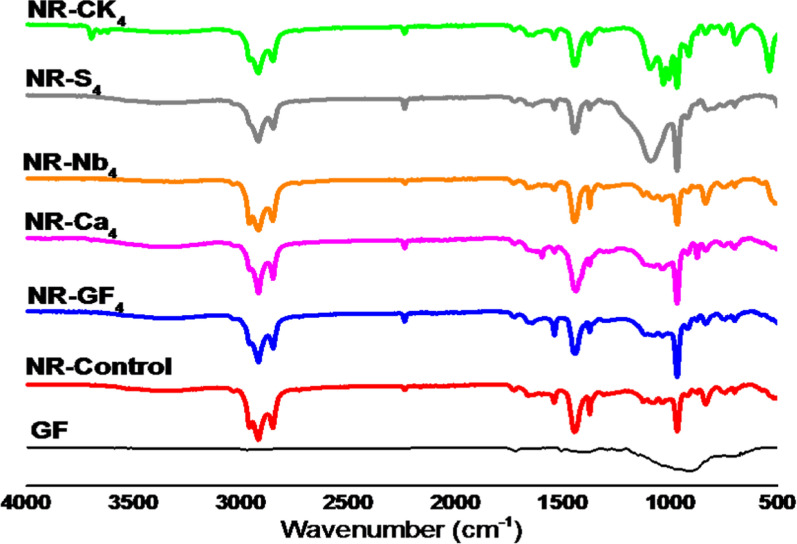
FTIR spectra of the glass fiber and NR composites

Figure 2 presents the characteristic FTIR peaks of glass fiber and NR composites with different filler loadings. A significant spectral peak in the glass fiber at 909 cm^**−1**^ is attributed to the stretching mode of the oxygen-silicon bond (Si–O–Si) in the glass fiber used; the Si–OH bending band was most likely the one that was found at 771 cm^**−1**^. Other oxides, such as boron oxide and aluminum oxide, contribute to a peak at 1434 cm^**−1**^. FTIR spectra of pure NR without filler show peaks at 2959, 1661, 1446, 1375, and 835 cm^**−1**^ corresponding to C–H stretching, CH=CH stretching, C–H bending, CH_**3**_–CH_**3**_ deformation, and CH_**2**_=CH_**2**_ out-of-plane deformation, respectively [27]. In the composite spectrum, a reduction in the intensity of the peak at 835 cm^**−1**^ due to the presence of GF in the NR matrix. This change in intensity is suggests the chemical interaction of the GF to the NR chain. Additionally, in the NR composites, the C = C stretching vibration shifts from its typical position at 1661 cm^**−1**^ in pure NR to 1640 cm^**−1**^, indicating potential interaction between the rubber and filler. Regarding filler–rubber interactions, the inclusion of various fillers in NR matrix leads to an increased intensity of the peak at 967 cm^**−1**^ in the composite spectrum, confirming successful attachment of the fillers to the NR backbone chain. Notably, NR-CK_**4**_ exhibits two distinct peaks at 538 and 468 cm^**−1**^, corresponding to Si–O-Al bending and Si–O bending vibration, respectively.

### Microscopy

**Fig. 3:**
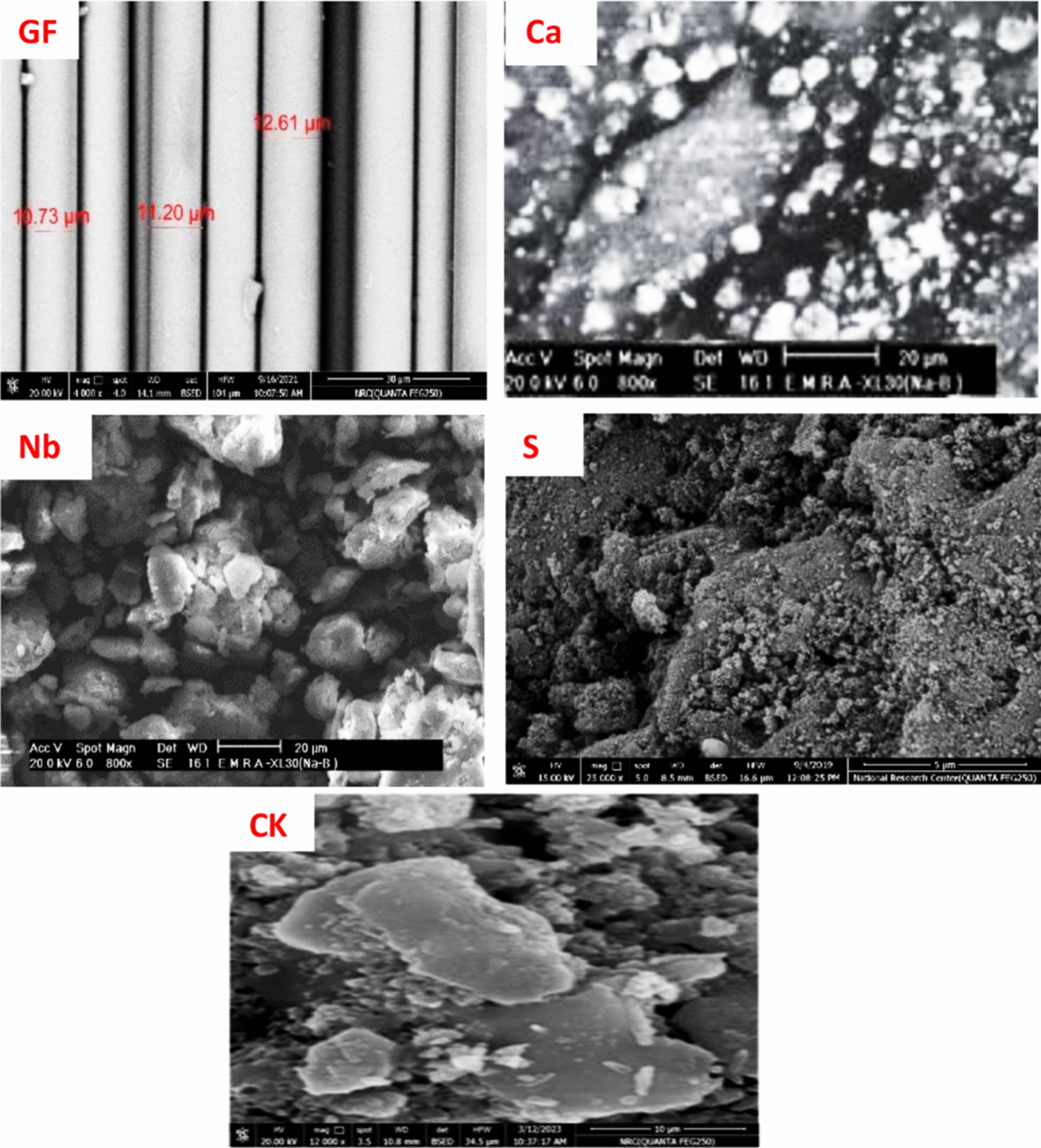
Micrographs of the fillers used. Glass fiber; CaCO_**3**_; Na-Bentonite; Silica; Calcined kaolin

To investigate the surface morphology of fillers and their distribution state in the NR matrix, SEM analysis was performed. From Fig. 3a, it is evident that GF exhibits a regular rod-like shape with particle sizes in the range of 10–12 µm. CaCO_**3**_ particles are spherical in shape, as illustrated in Fig. 3. Sodium bentonite (Nb) displays larger particle sizes, more amorphous crystals, and more disorder, with longer crystals and, to a lesser extent, crystals with defined geometry. In contrast, silica exhibits irregular geometries, including spherical clusters of cylindrical, flaky, angular porous, and spongy structures [28]. The calcined kaolin particles are observed as smooth-surfaced, layered sheets.

**Fig. 4:**
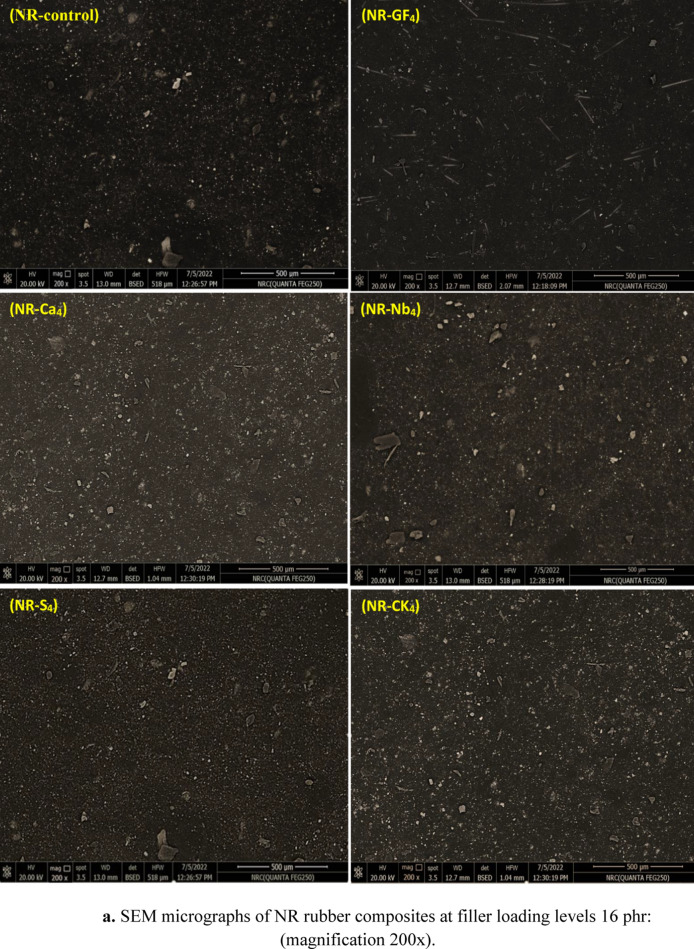

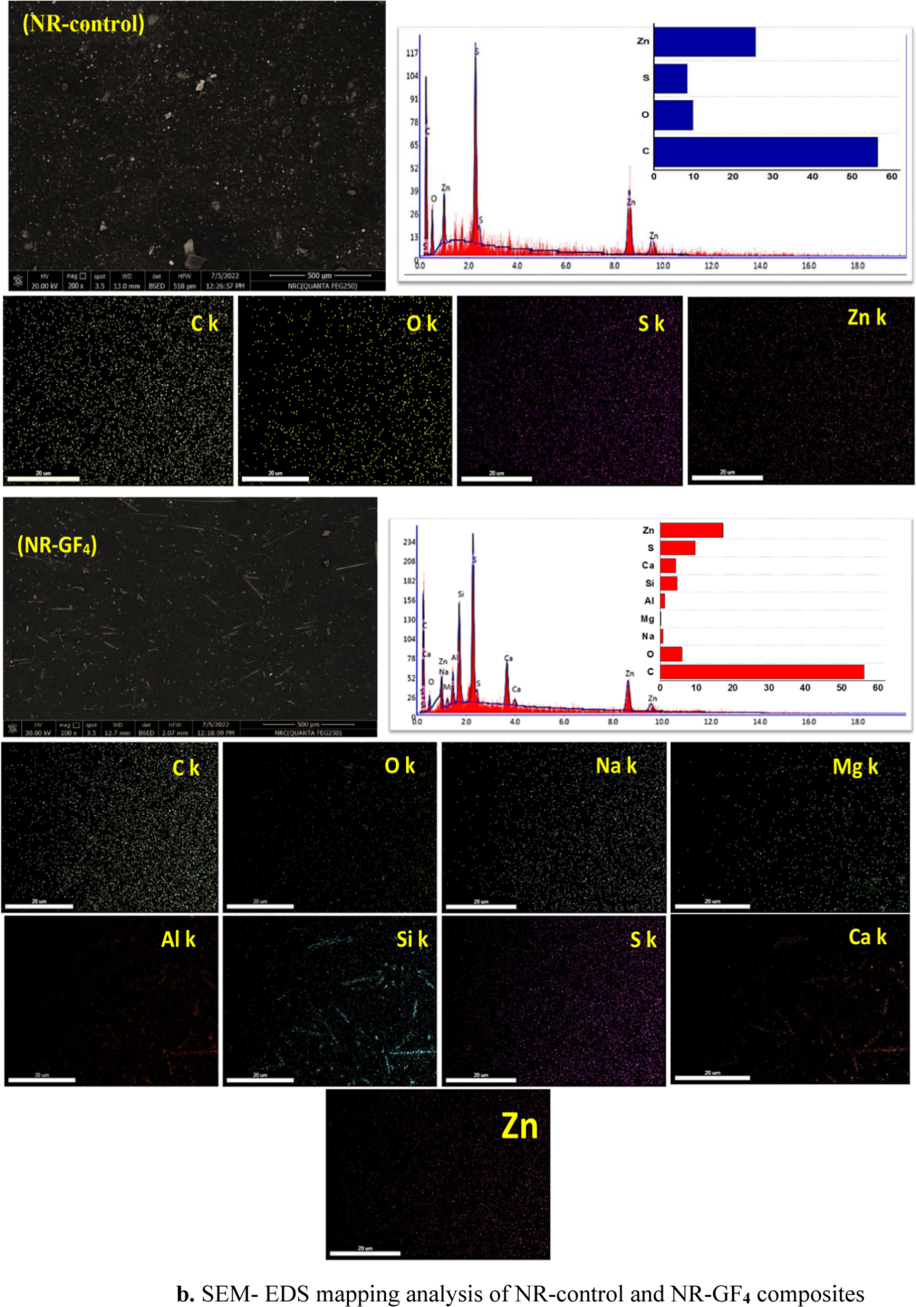
**a** SEM micrographs of NRrubber composites at filler loading levels16 phr: (magnification 200x). **b** SEM–EDS mapping analysis of NR-control and NR-GF**4**composites.

The tensile-fractured surfaces of NR composites with different fillers are presented in Fig. 4a. The performance of the composites improves when the fillers are uniformly dispersed; however, aggregation leads to stress concentration points, which in turn cause deterioration of the composite properties [29]. As seen in Fig. 4a, the NR-control surface was uneven and rough, and the addition of fillers slightly changed it. The small, visible white particles on the surface were zinc oxide particles. The loading of GF was connected to the smoothness of the tensile fractured surfaces for the NR-GF_**4**_ composites. Among the composites, the GF particles were well arranged in a rode-shaped structure, indicating strong adhesion between the NR macromolecular chain and the GF. The intercalated Na-bentonite was well dispersed with minimal agglomerates and exhibited good adhesion to the NR matrix. Similar behavior was observed for the calcined kaolin composites (NR-CK_**4**_). At higher filler loadings, expanded surface morphologies (NR-Ca_**4**_ and NR-S_**4**_) were observed, resulting from the accumulation of additional stress within the rubber matrix. The mechanical properties of the rubber composites were negatively affected by the agglomeration of silica and CaCO_**3**_ particles in the NR matrix, as this reduced the interparticle distances and increased filler-filler interactions relative to the filler-rubber interactions [30]. Moreover, the addition of the silane coupler (APTES) promoted dense interconnection between particles in all composites, demonstrating a high degree of particle interaction and facilitating the formation of a stable connection pathway. Overall, it can be concluded that, compared to other fillers under investigation, intercalated glass fiber exhibits superior compatibility with the NR matrix.

SEM–EDS mapping was conducted on NR-control and NR-GF_**4**_ composites (Fig. 4b). The elemental maps for the control sample (no filler) indicated that oxygen (O), carbon (C), sulfur (S), and zinc (Zn) elements were relatively uniformly distributed throughout the sample. The presence of S and Zn is attributed to ZnO and sulfur used during the vulcanization process. For NR-GF_**4**_, the maps revealed the distribution of both residues from the vulcanization stage and the glass-fiber reinforcement. Characteristic elements of glass fiber (GF), namely oxygen (O), silicon (Si), aluminum (Al), and calcium (Ca), were detected, along with trace amounts of sodium (Na) and magnesium (Mg). These results confirm that both the curing additives and glass fiber were uniformly distributed throughout the NR matrix, supporting the conclusion of effective filler-matrix bonding [10, 31].

### BET analysis

**Fig. 5:**
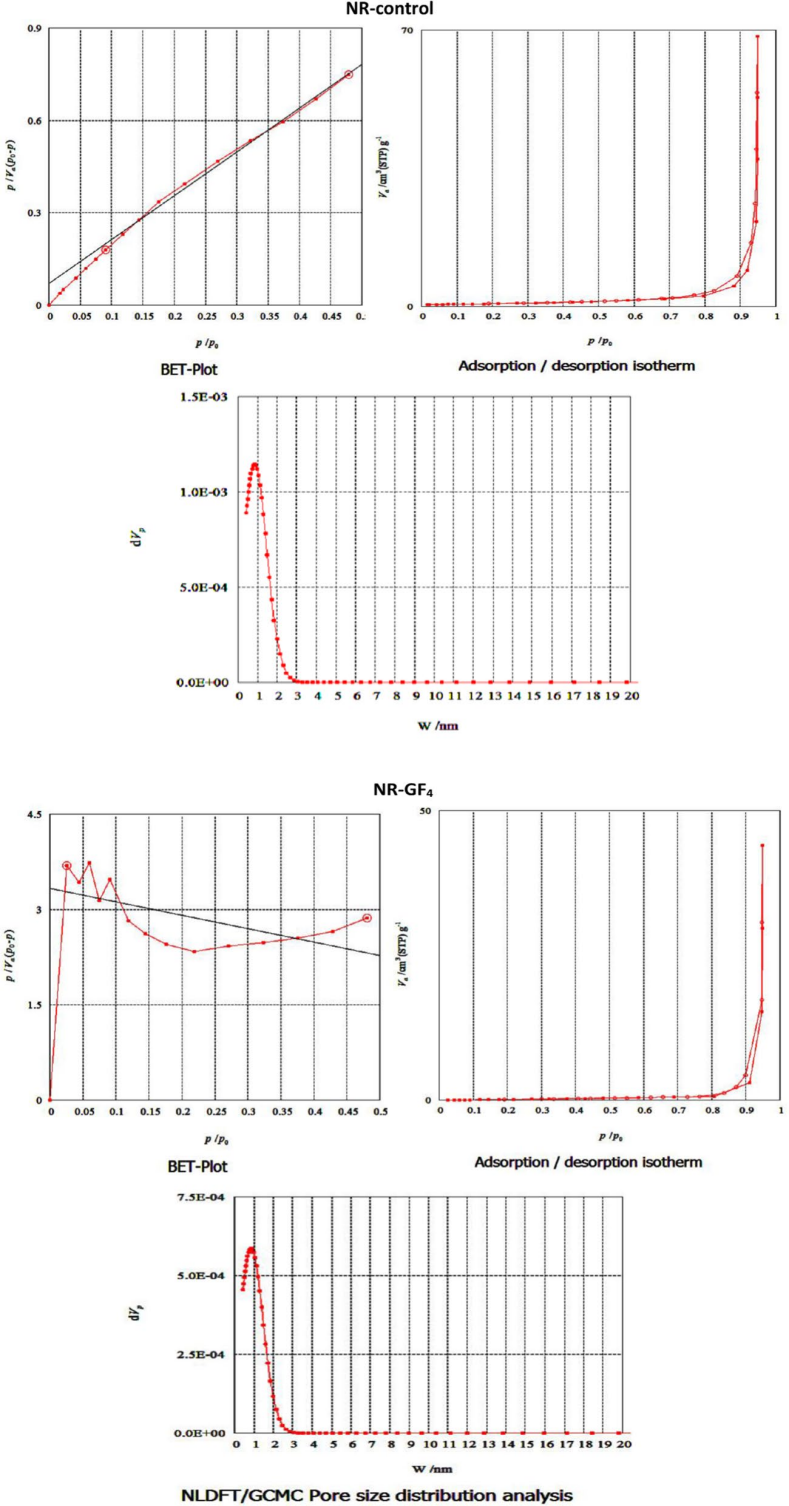
BET analysis of NR composites: surface area, pore volume, and pore size distribution

Figure 5 presents the BET analysis, highlighting distinct differences between NR-control (unfilled) and glass fiber (GF) reinforced NR composites (NR-GF_**4**_) to determine the specific surface area, pore volume, and pore size distribution. As summarized in Table 2, the NR-control matrix showed a BET surface area of 29.1 m^**2**^/g, a total pore volume of 0.106 cm^**3**^/g, and an average pore diameter of 14.5 nm. These values confirm the compact structure of unfilled NR, consistent with the relatively low amount of free volume and microvoids confined within the polymer chains. The addition of 16 phr glass fiber increased the surface area to 35.8 m^2^/g, which represents an increase of approximately 23% compared to the NR-control. Interestingly, the pore volume decreased significantly from 0.106 to 0.068 cm^3^/g (a reduction of ~ 36%)**,** demonstrating that the GF induced denser packing and lowered the overall porosity. In contrast, mean pore diameter increased considerably, from 14.5 nm in NR-control to 76.2 nm in the NR-GF_**4**_ composite, suggesting the formation of larger meso/macropores most likely associated with interfacial voids or partial fiber-NR matrix bonding [32]. The incorporation of GF into NR thus increases specific surface area, decreases total pore volume, and results in larger pore channels. These structural changes are expected to influence the reinforcement mechanism in NR, where enlarged interfacial voids may hinder stress-transfer efficiency, whereas the increase in surface area may enhance filler–matrix interfacial adhesion.

**Table 2:** BET surface area and porosity data for NR-control and NR-GF_**4**_ composites

Sample	BET surface area (m^2^/g)	Total pore volume (cm^3^/g)	Mean pore diameter (nm)
NR-control	29.1	0.106	14.5
NR-CK_4_	35.8	0.068	76.2

### Rheometric characteristics

Before starting the vulcanization process of rubber compounds, it is crucial to determine the cure kinetics in order to establish the precise conditions required for complete curing. Table 3 shows that all NR composites reached higher M_**H**_ and delta torque (ΔM) than the NR-control. The ΔM increased with increasing GF loading in NR composites. The explanation for this increase in ΔM can be attributed to several factors, including higher cross-link– density, the presence of rigid components that restrict the mobility of rubber chains, the reinforcing capacity of the fiber or filler, and interactions between filler and matrix. Due to the formation of a cross-linked matrix, GF exerts a beneficial effect on composite materials. GF improves the reinforcing efficiency of natural rubber, thereby influencing the vulcanization process and improving the matrix's ability to form cross-links. The minimum torque of NR composites also increased with filler loading. In this case, the silica particles in all investigated fillers increase the available surface area for cross-link formation during vulcanization. Consequently, the minimum torque serves as an indicator for the viscosity and mechanical properties of the NR composites. Analysis of the optimum cure time (T_**C90**_) and scorch time (T_**S2**_) demonstrates that both the type and concentration of filler affect curing properties. Specifically, all fillers interfered with the vulcanization process of NR composites. From Table 3, scorch time decreased with the addition of the investigated fillers present in the formulation [33]. A reduction in scorch time allows for faster processing, enabling manufacturers to produce more parts in a given timeframe.

**Table 3:** Vulcanization characteristics of the NR composites studied

Sample	M_H_, dNm	M_L,_ dNm	Torque difference, dNm	Tc_90,_ min	Ts_2,_ min
NR-control	11.53	0.9	10.63	18.35	4.6
NR-GF_1_	10.12	1.12	9.0	15.36	1.93
NR-GF_2_	11.58	1.25	10.33	14.71	1.81
NR-GF_3_	11.58	1.51	10.07	15.11	1.84
NR-GF_4_	13.13	1.33	11.8	12.64	1.35
NR-Ca_1_	11.3	1.32	9.98	13.96	1.63
NR-Ca_2_	11.34	1.49	9.85	13.68	1.68
NR-Ca_3_	12.38	1.62	10.76	16.45	1.62
NR-Ca_4_	11.71	1.34	10.37	15.07	1.61
NR-Nb_1_	12.07	1.27	10.8	14.02	2.43
NR-Nb_2_	13.39	1.44	11.95	12.99	2.7
NR-Nb_3_	10.77	1.24	9.53	16.81	3.8
NR-Nb_4_	11.22	1.58	9.64	17.11	3.79
NR-S_1_	11.14	1.19	9.95	15.62	2.26
NR-S_2_	11.23	1.42	9.81	16.28	2.24
NR-S_3_	12.47	1.72	10.75	15.34	2.23
NR-S_4_	12.9	2.47	10.43	14.53	2.17
NR-CK_1_	11.46	0.99	10.47	16.51	1.87
NR-CK_2_	9.35	0.91	8.44	16.97	2.41
NR-CK_3_	11.91	1.35	10.58	15.21	2.25
NR-CK_4_	12.87	1.48	11.39	15.67	2.22

Hence, the reduction in scorch time with increasing GF loading can be explained by the combined effect of all silica (SiO_**2**_) and other metal oxides (calcium, magnesium, and aluminum) present in the structure of GF. These oxides increase the availability of reactive sites and accelerate the cross-linking between rubber chains, resulting in a quicker onset of curing, as reflected in the shorter scorch time. A similar decrease in optimum cure time was observed in all NR composites. The NR/GF exhibited shorter optimum cure times compared to other composites, owing to the higher GF loading, which introduced more SiO₂ and metal oxide particles into the NR matrix. The oxide particles acted as activators during vulcanization, thereby accelerating the initiation of cross-linking. Although the oxide particles increase the scorch temperature, they also promote a faster rise to this temperature, resulting in a shorter scorch time than other composites [34].

### Mechanical properties

**Fig. 6:**
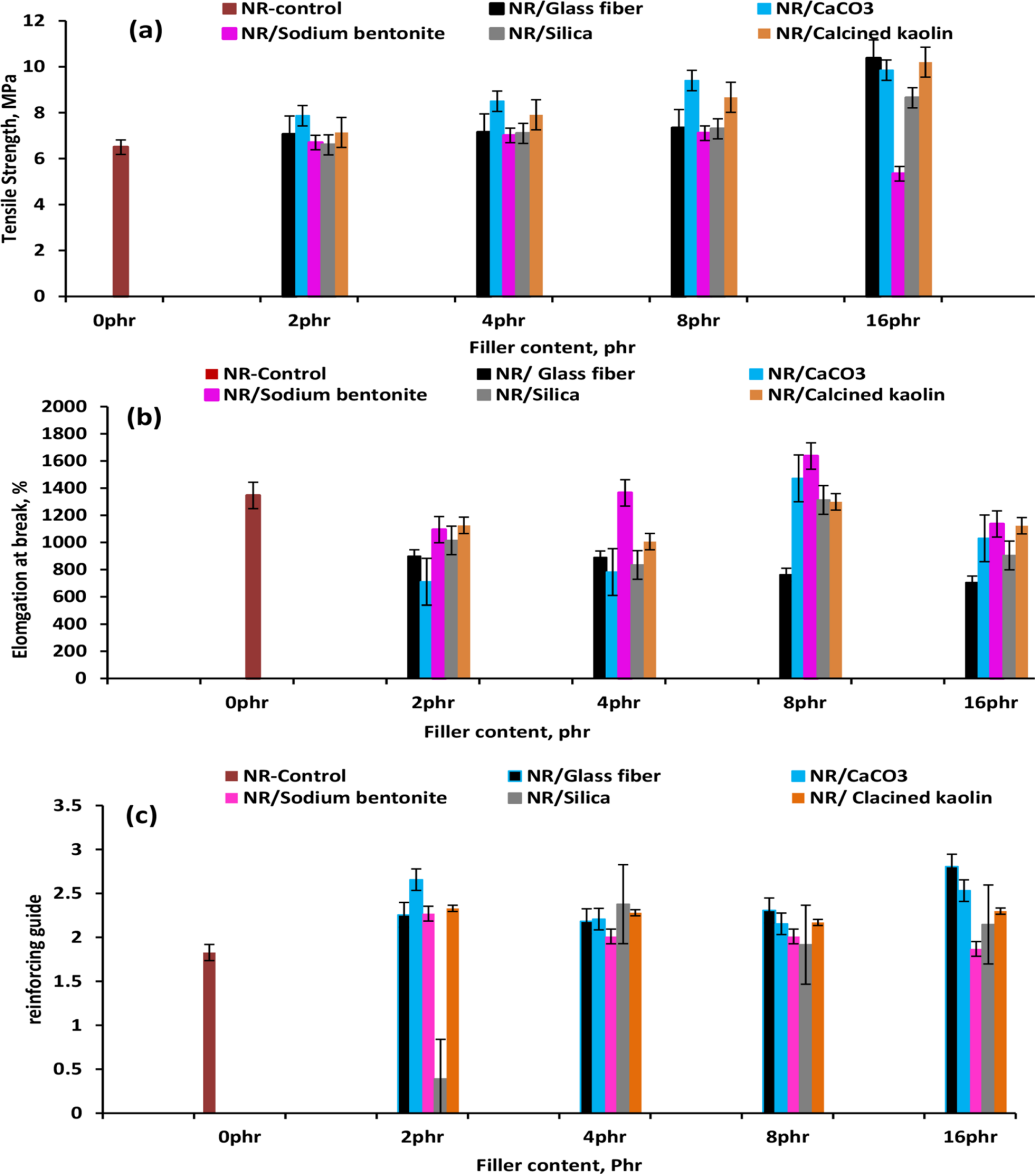
The mechanical properties of NR composites **a** Tensile strength, **b** elongation at break and **c** reinforcing guide

This study demonstrates that incorporating various fillers into a natural rubber (NR) matrix is a viable strategy for developing composites with enhanced multifunctional properties. Figure 6a–c shows the tensile strength, elongation at break, and reinforcing guide (reinf. Guide = (mod.at 300%)/(mod.at 100%)) (neat natural rubber (Neat NR); the NR/GF composite; the NR/Nb composite; the NR/S composite; and the NR/CK composite), these results prove that the outline of diverse fillers is a promising scheme to donate the natural rubber medium compounds with multi-functional features. That is one evident such adding fillers enhances natural rubber's mechanical properties. Strong interfacial interaction and effective stress transfer from the natural rubber matrix to the studied fillers were anticipated. This is especially in the NR/S composite, where the functional groups are anchored on the silica surface, forming some strong chemical bonds with the natural rubber chains in. Furthermore, the glass fiber exhibited the ability to entangle with natural rubber chains, further improving rubber matrix composites. The reinforcing guides (reinf. Guide) (Fig. 6c) were used to assess the reinforcing effect of investigated fillers and glass on the natural rubber matrix. Figure 6c represents the modulus ratio of 300% strain to 100% strain. Higher reinforcing guide values indicate a stronger reinforcing effect on natural rubber matrix reinforcement [34].

Specifically, the NR/S and NR/GF composites had the highest reinforcing guide values. The results demonstrated that the NR matrix containing Si or GF showed superior reinforcing capabilities due to the addition of a silane coupling agent to the fiber or silica surface, which allows the Si and GF to be firmly bonded to the natural rubber surface. As a result, the entanglement of NR molecular chains with fiber or fillers provided anchoring sites, enhancing the filler-natural rubber interface interaction [35]. The inclusion of GF significantly enhanced the virgin NR matrix's tensile strength. This can be attributed to the outstanding interactions between the natural rubber matrix and the GF surface, although it was accompanied by a reduction in the elongation at the break. This decline in the ductility demonstrates that adding GF into the NR matrix reduces flexibility as reinforcement increases. These findings are consistent with the study by Manaila E et al. [7], who developed a new natural rubber (NR) composite using hemp fibers instead of conventional fillers like carbon black or silica. They observed increased hardness and tensile strength properties with higher fiber content, indicating effective reinforcement due to strong fiber-rubber interaction. The restricted molecular chain motion and the homogeneous dispersion of filler in the rubber matrix are crucial for enhanced mechanical properties [36]. The filler particles present between the macromolecular chains allow the chains to dissipate strain in deformation by sliding across one another [35, 37]. However, not all filler exhibited this behavior. Increasing loading of CaCO₃ or Nb beyond 8 phr resulted in a small decrease in tensile strength and elongation at break. This decrease in the reinforcing effect is attributed to particle aggregation within the matrix after optimal dispersion (8 phr) has been exceeded. The effect of GF and different filler loadings on elongation at break is depicted in Fig. 6b. It was found that the elongation at break diminished due to the interfacial connection among the GF or different investigated fillers and the NR rubber matrix, as well as the improved interaction of the GF or different investigated fillers inside the rubber matrix, which plays a key role in decreasing the elongation at break. The enhanced interfaces between the GF or other investigated plaster and the natural rubber matrix improved the composites’ strength and toughness but reduced their flexibility [38]. In conclusion, it is evident that there is a clear strength-ductility trade-off: the composites with the greatest tensile strength (NR/S and NR/GF) also exhibited the lowest elongation at break. This opposite relationship is well established in materials science and is a popular rule of thumb for measures that improve a material's strength and reduce ductility.

### The stress–strain behavior of NR/GF or NR/ different types of filler composites

**Fig.7:**
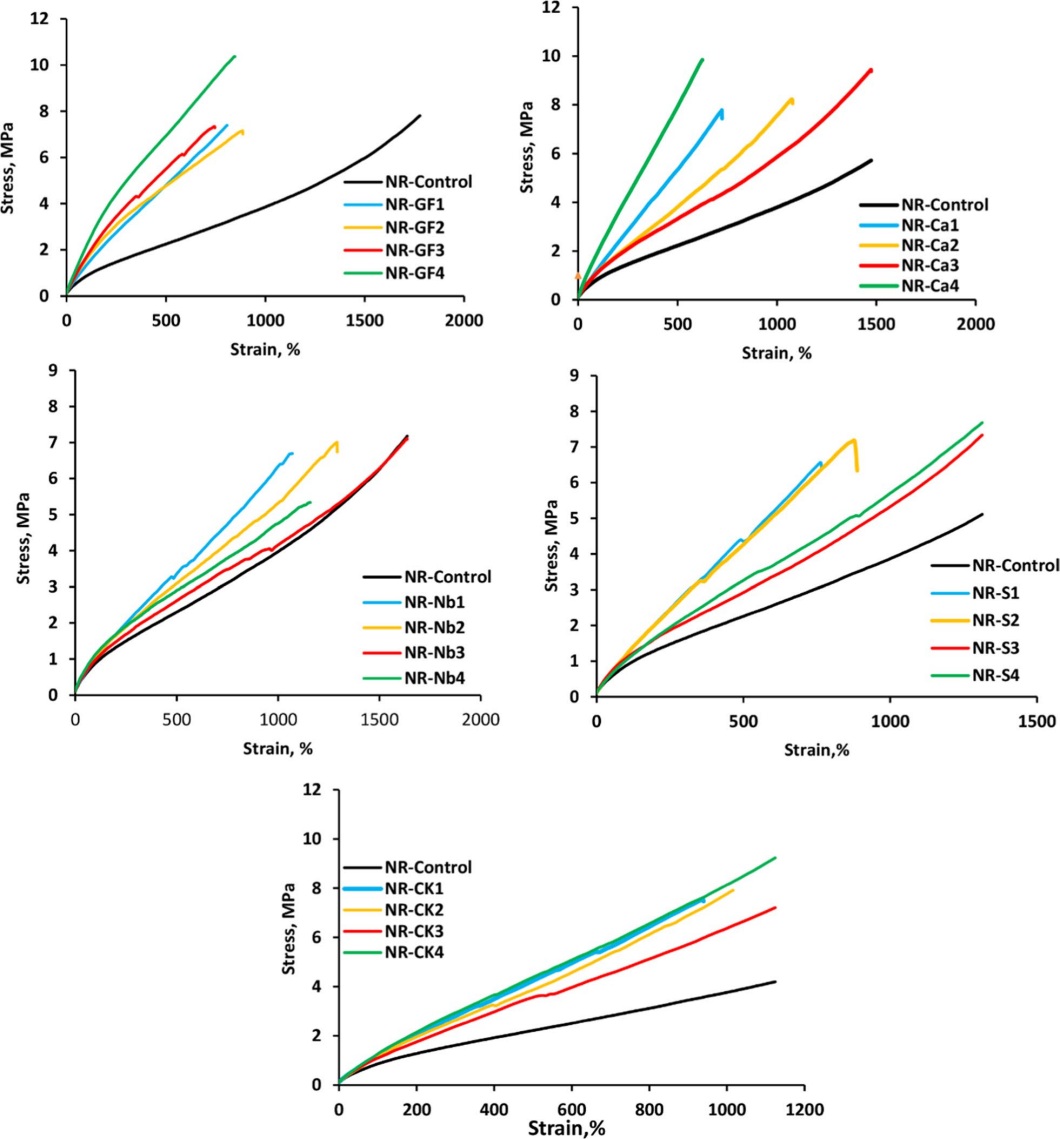
Stress–strain curves for natural rubber filled with various investigated fillers loading

The stress–strain properties are an important characteristic for rubber vulcanizates. The filler properties, which include atom size, surface activity, surface area, aggregate structure, and rubber-filler interactions, are primarily responsible for the dispersion state of the filler particles through the rubber matrix and consequently determine the degree of reinforcement [39]. Another significant factor in the reinforcing effect is the interaction-either physical or chemical-between the rubber chains and the filler particles [40]. Figure 7(a-e) shows the stress–strain curves for NR vulcanizates loaded with various concentrations of GF or fillers. These curves are typical of elastomeric materials, exhibiting an extended plastic flow area and an unclear yielding point. The kinetic hypothesis is valid at low stresses. A stress-softening effect is obtained at moderate strains at big deformations. Conversely, the addition of GF or fillers increases the tensile stress. Maximum stress values were obtained at 16 phr for all investigated fillers, except for Na-bentonite, which has a maximum stress value at 8 phr. This behavior may be attributed to the higher cross-linking density achieved in NR at that concentration. Overall, both the type and concentration of loaded filler were found to significantly affect the stress–strain behavior.

**Fig. 8:**
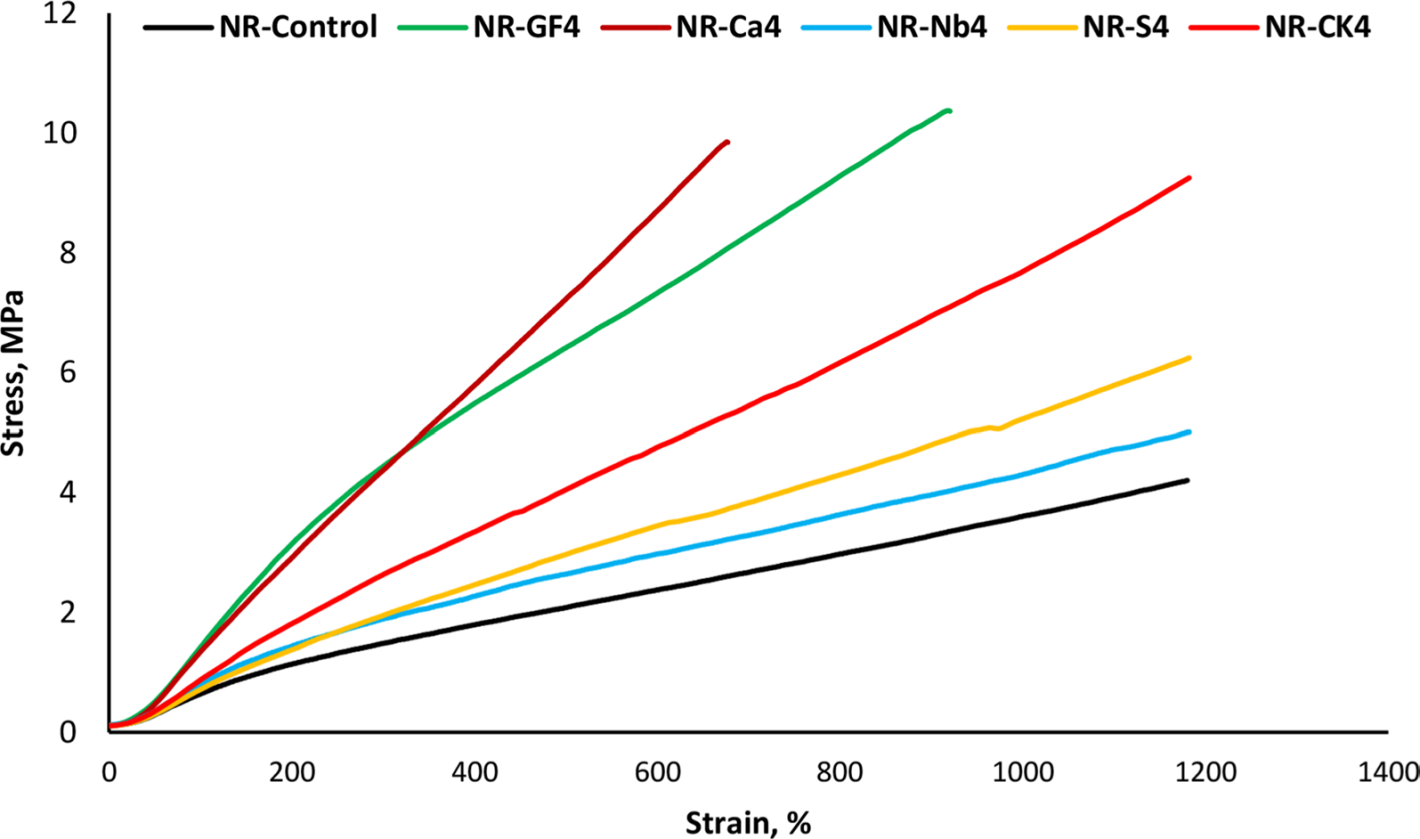
Comparative stress–strain curves of natural rubber filled with various investigated fillers loading (16phr)

Figure 8 shows the stress–strain curve of NR loading with 16 phr of different types of fillers. It is observed that the NR/Na-bentonite composite exhibited the lowest stress and did not perform well under the applied conditions, suggesting that Na-bentonite should be combined with others to improve reinforcement. When 16 phr of CK, CaCO_**3**_, or Si was added, the stress of rubber composites increased markedly, owing to the good dispersion of filler particles within the NR matrix. Otherwise, the addition of 16 phr of GF resulted in a slight increase in the stress due to interfacial adhesion, limited agglomeration, and good attachment between GF and the NR chain, which is responsible for the improvement of vulcanizate properties. Consequently, the strengthening effect of GF at the NR-filler interfacial interface was evident, influencing the stress behavior of the composites.

Glass fiber and the other fillers incorporated in elastomeric blends have a significant influence on the dynamic and performance of the prepared vulcanizates. The Payne effect evaluates filler-filler interactions in the composites through the complex modulus (G*) softening on small strain testes, which are on the linearity zone of an empty elastomer and therefore is an event that should not occur with the rubber chains only [41, 42].

**Fig. 9:**
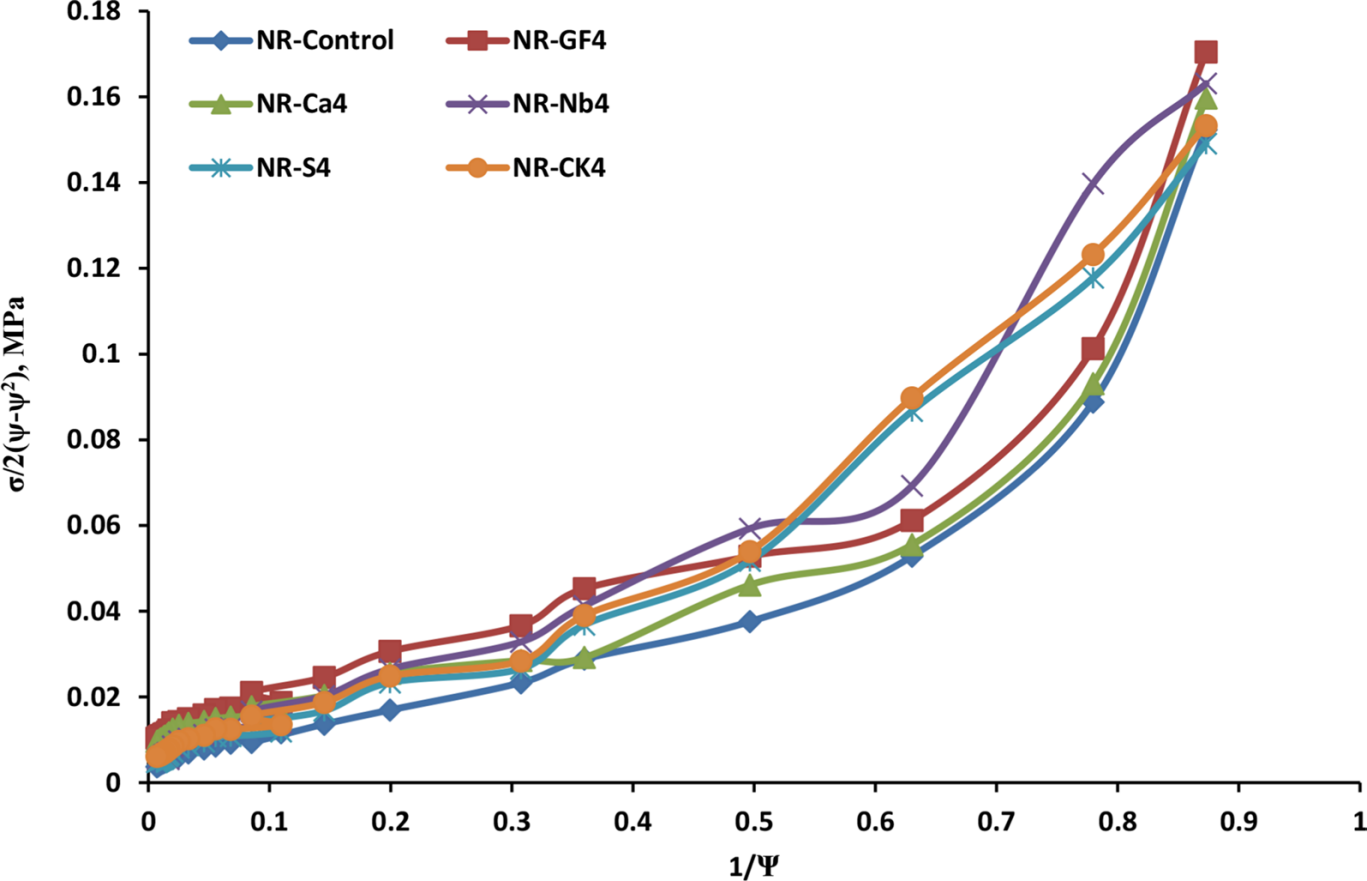
Mooney-Rivlin curves of of natural rubber filled with various investigated fillers loading (16phr)

The stress–strain behaviors were represented in Mooney-Rivlin plots in Fig. 9. The Mooney-Rivlin [43, 44] equation is given by;

8 $$\frac{\sigma }{2( \psi - {\psi }^{-2})}={C}_{1}+ {C}_{2}{\psi }^{-1}$$

wherein C_**1**_ and C_**2**_ are constants that represent the distinctive characteristics of NR rubber vulcanizates, σ is the tensile stress, and ψ is the strain. In NR composites, C_**2**_ denotes the non-Gaussian characteristics of the network and parameters, while C_**1**_ is an amount related to the best flexible behavior, and C_**2**_ shows the deviation after it. According to Fig. 9, increasing as fiber loading reduced the upturn strain. The obtained data suggest that interactions occur between the NR chain and GF or other fillers. The Mooney-Rivlin constant C_**2**_ was linked to the flexibility of NR rubber chains, as shown in Table 4. This value increased with the loading of Si, GF, calcium carbonate, Nb, and CK, respectively, reflecting filler–filler interaction. Higher C2 values, particularly in the NR/GF and NR/CaCO_**3**_ composite chain, indicated stronger chain entanglement within the NR matrix.

**Table 4:** The value of constant C_**1**_ and C_**2**_ for NR composites

properties	NR-neat	NR-GF_4_	NR-Ca_4_	NR-Nb_4_	NR-S_4_	NR-CK_4_
C_1_, MPa	0.0100578	0.0136415	5.5386 × 10^**–3**^	0.010876	0.0102146	0.014977
C_2_, MPa	0.055624	0.080638	0.080357	0.05966	0.074969	0.07729

Higher chain entanglement was associated with improved molecular-level mixing. As a result, the Mooney-Rivlin equation and the observed mechanical property fluctuation can be closely related. Conversely, fillers such as GF and CaCO_**3**_ were evenly distributed in NR vulcanizates based on the C_**2**_ value. As can be seen in Fig. 9, the upturns at greater stresses clearly illustrate the reinforcing effect for both the filled and unfilled samples [45]. In the unfilled samples, this upturn arises from one of its most distinctive features: its ability to crystallize under strain. This phenomenon, referred to as strain-induced crystallization, is mostly to blame for the sharp increase stress at high deformation. These results are consistent with the reinforcing guide values and SEM morphological observations [46].

### Swelling resistance

The results obtained for all the samples indicate that the rubber composites exhibit excellent resistance to water. No water absorption or water adsorption was observed on the sample surfaces. The results support the fact that the natural rubber composite produced are suitable for automotive accessories that are frequently exposed to water during washing. The addition of glass fiber (GF) or other investigated fillers in natural rubber composites imparts advantageous properties. The key filler characteristics, particle size, surface area, surface activity, aggregate structure, and nature of NR-filler interactions, depend primarily on the dispersion state of filler particles [47]. The swelling percentage and cross-linking densities of the prepared NR vulcanizates are presented in Table 5. As the content of GF or other fillers increased, the swelling ratio decreased. This trend reflects the well-established inverse relationship between the swelling percentage and the cross-linking density of the polymer network [48]. Table 5 demonstrates that the swelling ratio of the composites was reduced when the amount of GF or fillers increased [49]. The observed increase in the cross-linking density may also be due to the strong interfacial interaction between natural rubber and GF or fillers [48, 50]. This is anticipated to result from enhanced interactions between the natural rubber and the filler as well as enhanced cross-linking of NR chains intercalated between the GF or filler interlayer gap. The restricted mobility of the rubber chains in such confined regions contributes to higher cross-linking density and reduced equilibrium swelling, particularly at 16 phr of GF or other fillers. Accordingly, these materials demonstrate excellent durability under humid or aqueous conditions. The interactions between NR and fillers were further analyzed using the Lorenz and Parks equation [22].

**Table 5:** The influence of NR/investigated different of filler composites on the swelling characteristics

Sample numbers	ν_dens_, (mol/cm3)	Mole._C_ (g mol−1)	$\frac{{\text{EQ}}_{\text{f}}}{{\text{EQ}}_{\text{NR}}}$	$\frac{1}{{\text{EQ}}_{\text{s}}}$	ΔG, (J/mol)	ΔS (J/mol.K)	A(slope)	B(intercept)
NR -control	3.595 × 10^–4^	1391	**–**	0.5780	− 92.0383	0.30885		
NR-GF_1_	4.934 × 10^–4^	1013	0.8324	0.6944	− 127.628	0.42828	2.01392	− 1.1301
NR-GF_2_	5.114 × 10^–4^	978	0.8150	0.7092	− 132.354	0.44414		
NR-GF_3_	6.096 × 10^–4^	820	0.7341	0.7874	− 158.450	0.53171		
NR-GF_4_	6.884 × 10^–4^	726	0.5821	0.8475	− 179.357	0.60187		
NR-Ca_1_	5.111 × 10^–4^	978	0.8150	0.7092	− 132.354	0.44414	0.88327	− 0.04318
NR-Ca_2_	5.303 × 10^–4^	943	0.7980	0.7246	− 137.458	0.46127		
NR-Ca_3_	5.368 × 10^–4^	931	0.7973	0.7299	− 139.127	0.46687		
NR-Ca_4_	6.605 × 10^–4^	757	0.6994	0. 8264	− 144.690	0.48554		
NR-Nb_1_	4.254 × 10^–4^	1175	0.9075	0.6369	− 109.564	0.36766	0.3252	0.5644
NR-Nb_2_	4.708 × 10^–4^	1062	0.8555	0.6757	− 121.607	0.40808		
NR-Nb_3_	4.725 × 10^–4^	1058	0.8538	0.6770	− 122.052	0.40957		
NR-Nb_4_	4.763 × 10^–4^	1050	0.8497	0.6803	− 123.125	0.41317		
NR-S_1_	4.601 × 10^–4^	1087	0.8671	0.6667	− 118.789	0.39862	0.7906	0.08921
NR-S_2_	5.051 × 10^–4^	990	0.8208	0.7042	− 130.784	0.43875		
NR-S_3_	5.238 × 10^–4^	954	0.8035	0.7194	− 135.463	0.45457		
NR-S_4_	5.643 × 10^–4^	886	0.7688	0.7518	− 146.476	0.49153		
NR-Ck_1_	2.993 × 10–4	1670	1.1098	0.5208	− 76.0842	0.25532	1.8964	− 0.81678
NR-Ck_2_	3.823 × 10^–4^	1308	0.9653	0.5988	− 98.0762	0.32911		
NR-Ck_3_	4.545 × 10^–4^	1099	0.8728	0.6623	− 117.399	0.39396		
NR-Ck_4_	4.934 × 10^–4^	1013	0.8324	0.6944	− 127.399	0.39396		

υ_**dems**_ is defined as cross-linking density

9 $$\frac{{EQ}_{f}}{{EQ}_{NR}}=A{e}^{-Z}+B$$

10 $$\frac{{EQ}_{f}}{{EQ}_{NR}}=\frac{{V}_{r NR}(1-{V}_{rf})}{{V}_{rf}(1-{V}_{r NR})}$$

${V}_{rf}$ is the volume fraction (capacity tiny part) of the studied fillers occupying the NR phase in the swelling gel, ${V}_{rNR}$ represents the volume fraction of the pure gum NR, and Z is the ratio by weight of filler to NR hydrocarbon in the vulcanizate. E_**Q**_ is defined as the number of grams of solvent absorbed per grams of hydrocarbon. A and B are constants.

So that,

11 $$\frac{{V}_{r NR}}{{V}_{rf}}= A{e}^{-Z}+B$$

**Fig. 10:**
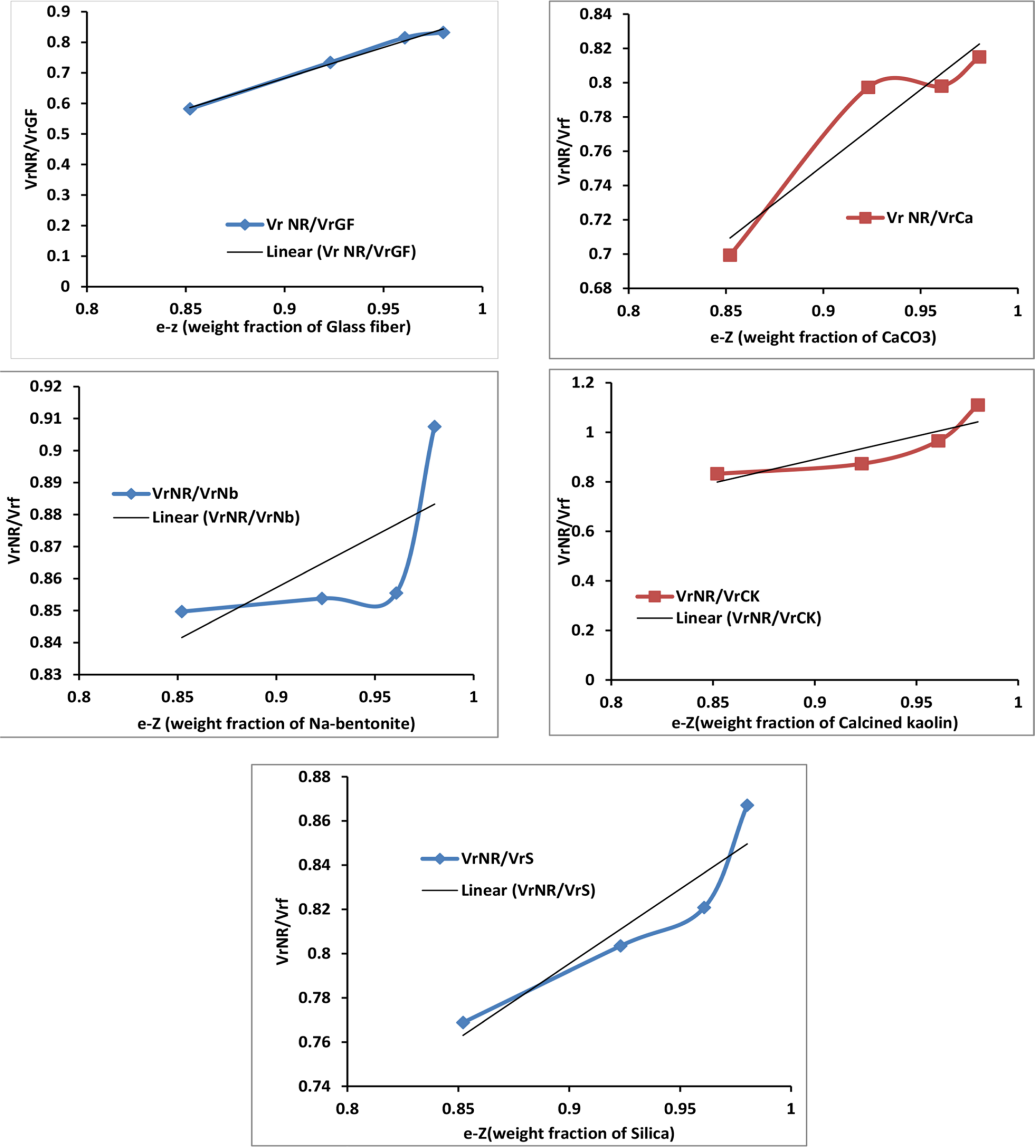
The variation between e–z (the weight fraction of investigated filler) and VrNR/Vrf (the volume fraction of control and filled rubber)

The values of A (slope) and B (intercept) were determined by plotting $\frac{{V}_{r NR}}{{V}_{rf}}$ (volume fraction of pure and filled rubber) against e^−z^ (weight percent of examined filler), as shown in Fig. 10. According to El-Sabbagh et al. [48], the reinforcement increases with a higher of slope value (A). The slope values are shown in Table 5, and the reinforcing abilities of filled NR composites can be arranged in the following order.

NR/GF˃NR/CK˃NR/Ca˃NR/S˃NR/Nb

As shown in Fig. 10, NR filled with GF exhibited a pronounced barrier effect compared to other filled NR composites. This behavior is due to the increased amount of GF acting as a reinforcing agent in the NR matrix, which restricts the NR rubber chains' ability to expand as a result of swelling. Consequently, toluene penetration between rubber molecules became more difficult, thereby reducing the swelling percentage. According to Abdelsalam et al. [49], the interaction between NR and GF or other fillers can be calculated using the parameter (1/EQs). Stronger filler-rubber interactions are indicated by higher values (1/EQs) and lower values of $\left(\frac{{\text{EQ}}_{\text{f}}}{{\text{EQ}}_{\text{NR}}}\right).$ The results in Table 5 show that NR composites containing 16 phr of GF (NR-GF_4_) demonstrated the highest (1/EQs) value and the lowest $\left(\frac{{\text{EQ}}_{\text{f}}}{{\text{EQ}}_{\text{NR}}}\right)$ ratio, confirming the strongest interaction between NR and GF. These findings are consistent with the Flory-Rehner connection equation [20], which relates the equilibrium swelling behavior to cross-link formation through which the molecular weight between cross-links (Mc). The obtained cross-linking densities (ν_**dens**_) and average molecular weights between two successive cross-links (Mc) are listed in Table 5. From equilibrium swelling and Mc data, it was determined that the extra physical and chemical cross-links were responsible for the increase in cross-linking densities ν_**dens**_ in the presence of GF or other fillers. Moreover, higher content of GF (16 phr, GF_**4**_) enhances these effects by serving as a bridging filler to connect with the NR matrix [49]. As the filler content increased, the Mc values of the composites decreased. This trend is attributed to higher crosslinking densities, and a greater fraction of the rubber matrix became non-swellable. Swelling resistance is a critical property for automotive applications, where rubber components frequently come into contact with fluids such as fuel, oil, and coolant. Exceeding critical swelling limits can lead to mechanical performance degradation and, ultimately, leakage or failure of components, affecting durability and reliability [51].

### Thermodynamic parameters

The thermodynamic characteristics of NR composites were used to investigate the dispersion of GF and other fillers within the NR matrix. The variations in elastic Gibbs free energy (ΔG) and conformational entropy (ΔS) for NR containing different filler concentrations are presented in Table 5. The elastic Gibbs free energy (ΔG), which serves as an indicator of material elasticity, exhibited a more negative value for the NR-GF_**4**_ composite (16 phr GF), indicating higher elasticity compared to other filler concentrations. Similarly, NR-GF_**4**_ composites displayed the highest conformational entropy (ΔS), which is primarily attributable to the homogeneous dispersion of GF_**4**_ within the NR rubber matrix. The data clearly show that a greater negative shift in (ΔG) corresponds to the increase in the thermodynamic compatibility between the filler and the matrix. A negative ΔG (ΔG < 0) indicates a thermodynamically stable system. For the NR-GF_**4**_ composite, increasing GF (or other fillers) content resulted in more negative values, demonstrating improved thermodynamic stability. This behavior can be explained by the effect of filler loading on dispersed phase size and increasing interfacial area up to an optimal content [34].

Moreover, higher filler contents led to increased conformational entropy (ΔS), which indicates greater molecular disorder in the system. This increase in ΔS suggests enhanced compatibility and interaction between the fillers and the NR matrix. In particular, 16 phr loading contributed to increased system disorder, which suggested improved interfacial and thermodynamic miscibility between phases.

### Cytotoxic activity testing of different rubber vulcanizates

The cytotoxicity (cell viability) of all natural rubber (NR) vulcanizates containing different additives was evaluated using a human normal fibroblast cell line (BJ1), and results are summarized in Table 6. All tested vulcanizate samples were found to be non-cytotoxic to the BJ1 cells, confirming their biocompatibility and safety for potential human contact. Furthermore, cell viability examinations implied that the additions of fillers and additives into the NR matrix did not negatively affect the cell viability, confirming that the NR composites developed were non-cytotoxic. The cytotoxicity testing of the NR/glass fiber vulcanizates using human normal fibroblast cell lines (BJ1) confirmed their favorable safety properties, suggesting suitability for biomedical applications. These findings offer valuable insights for potential uses such as surgical meshes and wound dressings, owing to their high strength, exceptional biocompatibility, and potential for cost-effective, sustainable manufacture. Glass fiber contributes enhanced tensile strength, dimensional stability, and corrosion resistance, while natural rubber provides biodegradability and affordability. However, challenges remain due to the limited mechanical properties and water absorption of natural rubber, requiring further modifications of NR/glass fiber composites in order to improve their overall performance and compatibility with the rubber matrix for specific biomedical applications. These results provide a benchmark for optimizing both environmental sustainability and product performance.

**Table 6:** Cytotoxic activity of different rubber vulcanizates towards human normal Skin fibroblast [BJ1]

IC_50_ (µg/ml)	Sample no
N.A	NR-control
N.A	NR-GF_4_
N.A	NR-Ca_4_
N.A	NR-Nb_4_
N.A	NR-S_4_
N.A	NR-CK_4_
N.A	DMSO
N.A	Negative control

Where *N.A.* no activity

## Conclusion

In conclusion, sulfur-cured natural rubber (NR) composites were successfully prepared with GF and various fillers. The incorporation of 16 phr GF significantly improved the mechanical properties and swelling resistance of the composites. Morphological analysis confirmed the formation of strong interfacial bonding between the GF and NR matrix, which contributed to enhanced overall performance. Rheological studies showed reduced processing time with increasing GF concentration. The composites exhibited outstanding improvements in tensile strength, modulus at 100%, 200%, and 300% deformation, Payne effect, and cross-linking density. Furthermore, the NR/GF composites displayed reduced swelling, making them promising composite candidates for automotive applications. Cytotoxicity assessments using human fibroblast cell lines (BJ1) confirmed their non-cytotoxic nature, supporting their potential for sustainable, high-performance automotive interior applications. Future research should focus modifications of NR/glass fiber composites and on long-term aging studies to further evaluate durability and ensure reliable performance under service conditions.

## Data Availability

The datasets used and/or analyzed during the current study are available from the corresponding author on reasonable request.
